# Divergent Patterns of Metabolite Expression in Red Seaweeds (*Devaleraea mollis* and *Palmaria hecatensis)* Following Nitrate and Ammonium Supplementation

**DOI:** 10.3390/life15020143

**Published:** 2025-01-21

**Authors:** Schery Umanzor, Jae Woo Jung, Muriel Dittrich, Jang K. Kim, Patrick Tomco, Zachary C. Redman, Monica Brandhuber

**Affiliations:** 1 College of Fisheries and Ocean Science, University of Alaska Fairbanks, Juneau, AK 99801, USA; mcdittrich@alaska.edu; 2 Department of Marine Science, Incheon National University, Incheon 22012, Republic of Korea; wodn8743@inu.ac.kr (J.W.J.); jang.kim@inu.ac.kr (J.K.K.); 3 Department of Chemistry, University of Alaska Anchorage, Anchorage, AK 99508, USA; pltomco@alaska.edu (P.T.); zcredman@alaska.edu (Z.C.R.)

**Keywords:** metabolomics, Pacific dulse, protein synthesis, rhodophytes, seaweed aquaculture

## Abstract

This study explores species-specific metabolic responses to different nitrogen-rich formulations in *Devaleraea mollis* and *Palmaria hecatensis*, highlighting distinct adaptive strategies. We evaluated the effects of Von Stosch Enrichment (VSE, nitrate-only), F/2 (nitrate-only), and Jack’s Special (JS, nitrate and ammonium) on metabolic profiles. *D. mollis* exhibited elevated energy storage and growth-related metabolites, with JS enhancing creatine production for energy storage and regeneration, alongside increased DNA/RNA synthesis and cell division activity. This suggests *D. mollis* prioritizes rapid growth and energy demands, supporting broader ecological adaptability. Conversely, *P. hecatensis* showed higher expression of metabolites linked to amino acid metabolism and protein synthesis, indicating a focus on efficient nitrogen use for protein production, likely advantageous in low-light, high-turbidity conditions. Nitrogen sources significantly influence amino acid metabolism, with JS promoting broader amino acid production and VSE and F/2 stimulating specific metabolites. These species-specific metabolic patterns underscore the flexibility of *D. mollis* in energy use versus adaptations of *P. hecatensis* to protein synthesis pathways. These findings highlight species-specific nutrient formulations as essential for optimizing seaweed growth and metabolic traits in aquaculture.

## 1. Introduction

Seaweed aquaculture continues expanding as a maritime industry outside Asia [1]. As the industry grows, species diversification becomes increasingly essential for enhancing its long-term viability, competitiveness, and sustainability. In the United States, Maine and Alaska are leaders in kelp production [2]. However, diversifying beyond kelp to include other groups, such as red seaweeds, can mitigate potential risks associated with the emergence of disease, market fluctuations, and environmental changes, particularly in the context of climate change-driven events [3,4]. Similarly, employing various farming practices, both at sea and on land, can help increase the industry’s resiliency and provide means to tailor biomass quality for targeted markets [5].

Among red seaweeds, *Devaleraea mollis* (formerly *Palmaria mollis*) and *Palmaria hecatensis* are emerging as promising candidates for land-based cultivation due to their high protein content and adaptability to controlled environments [6,7]. While *D. mollis* has successfully been grown in various conditions across the Pacific Northwest, *P. hecatensis* is being explored as a novel species for indoor cultivation in Alaska [8]. Differences in specific growth rates (SGRs) and responses to environmental factors such as temperature, irradiance, and nitrogen sources between these species highlight their distinct ecological and physiological adaptations despite sharing similar life histories and habitats [9,10,11]. *D. mollis*, for example, exhibits higher SGRs in warmer temperatures, suggesting greater thermal tolerance, whereas *P. hecatensis* seems to be better adapted to cooler environments [8]. Dittrich and colleagues described that although SGRs were similar when using different nutrient sources, *D. mollis* showed differences in thalli color, with some treatment samples looking pale compared to others. No color differences were observed in *P. hecatensis*.

Understanding the metabolic responses of these red seaweeds to different nutrient conditions is important for optimizing their cultivation. Metabolomics, which involves the comprehensive analysis of metabolites within a biological system, provides a powerful approach to exploring how different nitrogen sources influence seaweed metabolism, growth, and overall performance [12]. Nitrogen is a key nutrient for seaweed development. It is typically supplied as nitrate (NO_3_^−^) or ammonium (NH_4_^+^) in cultivation systems. However, the effectiveness of uptaking and assimilating each nitrogen form is species-specific and can significantly impact growth rates and the production of essential metabolites [13].

Recent metabolomic studies on red seaweeds describe how nitrogen form influences metabolite profiles, including the production of proteins, pigments, and other bioactive compounds. For example, studies on *Gracilaria* spp. demonstrated that ammonium supplementation led to higher production of phenolic compounds while nitrate favored carbohydrate accumulation, suggesting that nitrogen forms can modulate the balance between carbon and nitrogen metabolism [14,15,16]. Similarly, research on *Palmaria palmata*, a species closely related to *D. mollis*, has shown that different nitrogen sources can significantly affect the levels of bioactive compounds, making these findings relevant for optimizing the cultivation of *D. mollis* and *P. hecatensis* [17,18,19].

In land-based cultivation, optimizing nutrient supplementation is crucial for maintaining high-quality biomass while reducing operational costs. Although von Stosch enrichment medium (VSE) is commonly used for red seaweed cultivation, its high cost has driven research into alternative, more cost-effective nutrient sources such as the land fertilizer Jack’s Special (JS) [20]. Recent studies on *D. mollis* and *P. hecatensis* have shown that JS, which contains both ammonium and nitrate, can produce comparable or even superior growth rates and biomass quality compared to VSE while significantly reducing costs [9].

Differences in protein and pigment concentrations observed in seaweeds grown with different nitrogen sources highlight the complex relationship between nitrogen form and metabolic activity, underscoring the importance of tailored nutrient formulations for each species [9,21,22]. Given these findings, the present study aims to assess how varying nitrogen sources, whether provided as NO_3_^−^ alone or a combination of NO_3_^−^ and NH_4_^+^, influence the metabolite profiles of *D. mollis* and *P. hecatensis*. We hypothesize that the metabolic performance of both species will differ in response to the nitrogen supplementation provided, offering valuable insights that contribute to broader efforts to develop lower-cost yet higher-quality red seaweed biomass.

## 2. Materials and Methods

### 2.1. Sample Collection and Acclimation

Biomass for *D. mollis* and *P. hecatensis* was collected in May of 2023 from Middle Bay, Kalsin Bay (57.6317° N, 152.4003° W), and Pasagshak Bay (57.4478° N, 152.4750° W) in Kodiak Island, Alaska, during peak biomass availability. Although specimens were collected from the same locations, *D. mollis* is a low intertidal species, while *P. hecatensis* is located higher on the intertidal zone. This difference in distribution ranges along the intertidal zone implies differences in optimum light regimes and temperature driven by tidal changes [8].

Approximately 10 kg of healthy thalli per species were manually harvested from the intertidal zone at low tide (−0.5 m MLLW). Only thalli with proliferations, minimal herbivory, and no visible signs of decay or bleaching were selected. These proliferations are irregularly-shaped secondary growth stemming from the main thalli or secondary branches (see [23,24] for differences in morphologies). The biomass was wrapped in seawater-moistened paper towels, packed in sealed plastic containers with gel ice packs, and transported in coolers to the Juneau Center, College of Fisheries and Ocean Sciences, University of Alaska Fairbanks. The transit time was kept under 24 h. Upon arrival, the thalli were placed in two independent 1000-L recirculating tanks (30 ± 1 ppt salinity, 4 ± 0.5 °C, 50 ± 10 µmol photons m^−2^ s^−1^, 12L:12D) for a 14-day acclimation period to ensure growing parameters were adequate for growth. The tanks were cleaned and replenished with filtered (1 µm) and UV-treated seawater every seven days. Vigorous aeration was provided to ensure the continued tumbling of the culture.

### 2.2. Experimental Treatments

After the 14-day acclimation, 90 proliferations from each species were carefully detached from different parental thalli using tweezers. Proliferations were rinsed with sterile seawater (SSW), cleaned with a Kimwipe to remove surface contaminants, and grouped in sets of three individuals, each with a biomass of approximately 0.01 g ± 0.006 g. Each group was then placed into 250 mL Erlenmeyer flasks (*n* = 5) filled with SSW (30 ± 1 ppt) enriched with either Von Stosch enrichment medium (VSE), F/2, or Jack’s Special 25-5-15 (JS) along with 2 mL/L germanium dioxide to control diatom growth (250 mg/L stock solution) [25]. The flasks were incubated in Conviron Growth Chambers (Conviron GE1000, Pembina, ND, USA). The culture conditions for *D. mollis* consisted of an 8 °C, 40 ± 10 µmol photons m^−2^ s^−1^ irradiance, with a 16L:08D light-to-dark cycle. A second growth chamber was used for *P. hecatensis* replicates, set at 8 °C, 100 ± 10 µmol photons m^−2^ s^−1^, with a 16L:08D light-to-dark cycle. Differences in light regimes obeyed the need to avoid fertility and subsequent spore release of *D. mollis*, and to maintain steady growth in both species ([8]; see their result for growth data). All replicates remained in the experimental conditions for 28 days. Aeration was maintained throughout the experiment, and the treatment media were refreshed every seven days to prevent nutrient depletion. After the 28-day experimental period was completed, samples were frozen at −80 °C to stop all metabolic activity. Seaweed samples were stored at −80 °C until extraction for metabolic analysis.

The nutrient sources were chosen based on their availability, cost, and formulation, particularly regarding nitrogen molarity (see Table S1 for formulation details). Von Stosch enrichment medium (VSE) is a reagent-grade solution (500 μM N, 100% NO_3_^−^) recommended for red seaweed cultivation, prepared following protocols from Redmond et al. [25] and Werner and Dring [26] for *Palmaria palmata*. F/2, primarily used for microalgae (500 μM N, 100% NO_3_^−^), is a liquid pre-mix requiring only dilution of Part A and B (130 µL per liter of seawater), as described by Guillard and Ryther [27]. Jack’s Special 25-5-15 (JS) is a commercial plant fertilizer (500 μM N, 57% NO_3_^−^ and 43% NH_4_^+^) in pellet form [26], which was dissolved in 1 L of MilliQ water (MilliporeSigma, Darmstadt, Germany). After dissolving 100 g of JS, the solution was filtered (0.2 µm) to sterilize and remove undissolved particles before use as an experimental treatment.

### 2.3. Metabolomics

Approximately 40 mg of seaweed tissue (range 0.29–0.59 mg) was transferred to pre-chilled Precellys (Bertin Technologies SAS, Montigny-le-Bretonneaux, France) tissue homogenization tubes (P000918-LYSK1-A.O.). Extraction solution (1.5 mL, 50% methanol, 30% acetonitrile, 20% water by volume, all LCMS-grade) was added to each tube. Samples were then shaken in a temperature-regulated Bertin Precellys 24 with a Cryolysis unit for 5 min at 6000 rpm at a maximum temperature of 0 °C. The previous step was repeated using new 1.5 mL homogenization tubes. Samples were then vortexed for 30 min at 4 °C, followed by centrifugation for 10 min at 12,213× *g* at 4 °C. An aliquot of each supernatant (300 µL) was transferred to new centrifuge tubes and lyophilized with a Centrivap (Labconco, Kansas City, MO, USA) at −4 °C overnight or until dry. Samples were stored at −80 °C until analysis, when they were reconstituted in LC/MS grade water (200 μL) and transferred to 2 mL autosampler vials with inserts.

Liquid chromatography–mass spectrometry LC/MS spectra were collected using a ThermoFisher Scientific (Waltham, MA, USA) Orbitrap Exploris 120 with Vanquish Flex Binary UHPLC and an ACE C18-PFP column (100 × 2.1 mm, 2 μm packing material). Quality controls were created from a pool of all seaweed samples, sampled five times at the beginning and end of the sample run for each polarity mode and every nine samples throughout the sample run. Sample blanks of LC/MS grade water were sampled five times at the beginning and end of each sample run for each polarity to negate the detection of any background metabolites.

MS1 analyses were collected in positive and negative modes (4100 V for positive mode, 3200 V for negative mode). A 12-min mobile phase gradient of water (A) and LCMS-grade methanol (B) was conducted as follows: 100% A for 4 min, ramped to 5% A from 4 to 8 min, held at 5% A from 8 to 12 min. The flow rate was 0.250 µL/min, followed by a 3-min column wash of 100% water at 0.40 µL/min. Raw spectra were processed in Compound Discoverer v3.3 and matched to an in-house *m*/*z* Mine reference library consisting of IROA (Ann Arbor, MI, USA) standards (MSMLS Kit) with a retention time tolerance of 0.2 s. The in-built metabolomics workflows were used for each respective polarity. Only confirmed metabolite IDs were included in subsequent statistical analyses (Figure S1). A limitation of this approach is that our workflow does not consider metabolites outside of our compound library, potentially overlooking pathways involving other metabolites. The IROA MSMLS library is a large, albeit not comprehensive, set of metabolites that provide confirmation at Level 1 confidence [28]. We elected to focus on these high-confidence assignments.

### 2.4. Data Analysis

Metabolite data were preprocessed (normalized by sum, Log10 transformed, and mean-centered) using MetaboAnalyst v. 6.0 (Xia Lab, Edmonton, AB, Canada) to facilitate comparisons among spectra. A Principal Component Analysis (PCA), an unsupervised chemometric technique, was used to diminish dimensionality and visualize sample clusters of similar metabolite expressions. The corresponding loadings plot identified the metabolites responsible for PCA group separation. A supervised Partial Least Squares-Discriminant Analysis (PLS-DA) was performed to maximize discrimination among sample clusters. PLS-DA model performance was cross-validated using Leave-One-Out Cross-Validation (LOOCV), with only models with Q2 > 0.6 considered an appropriate fit for the dataset. Variable Importance in Projection (VIP) scores were used to determine which metabolites were the primary drivers of variation in the PLS-DA. Two-sample *t*-tests (*p* < 0.05) were used when comparing among species, and ANOVAs (α = 0.05) followed by paired *t*-tests were used when comparing among nutrient media types, to determine statistical significance.

## 3. Results

**Figure 1:**
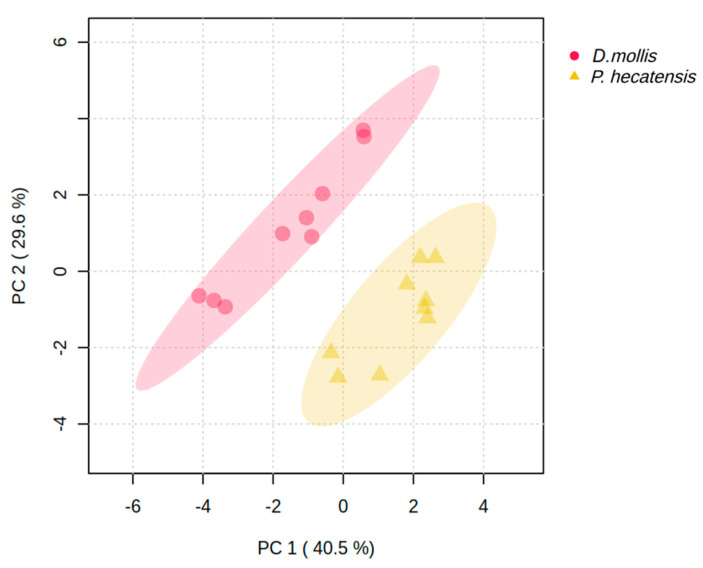
Principal Components Analysis (PCA) showing grouping differences between *Devaleraea mollis* and *Palmaria hecatensis* based on their metabolite profiles in response to nutrient supplementation with VSE, F/2, and JS (See Table S2 for statistical outputs).

Overall, metabolite profiles differentiated *D. mollis* from *P. hecatensis,* highlighting different metabolic strategies for incorporating, assimilating, and utilizing nutrients, particularly nitrogen (Figure 1).

*D. mollis* showed a higher capacity for energy storage and metabolic activity related to energy production. The species also showed higher RNA/RNA synthesis rates, aligning with greater cell division metabolite expression. *D. mollis* also showed higher expression of secondary metabolites linked to DNA synthesis and repair, biosynthesis of aromatic amino acids, and light sensing and UV protection. On the other hand, *P. hecatensis* showed greater expression of metabolites linked to protein synthesis and regulation (Figure S2, Table 1).

**Figure 2:**
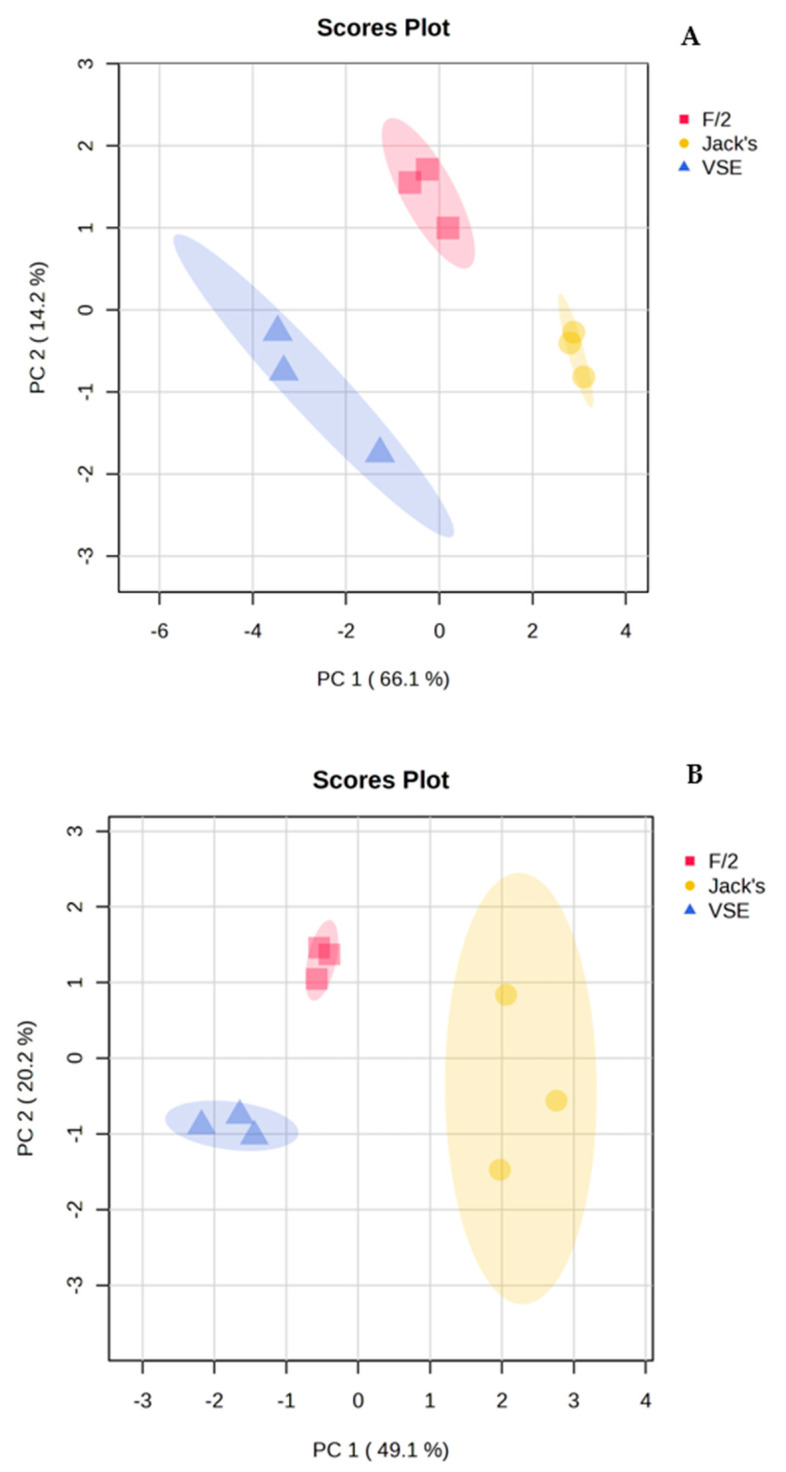
Principal Components Analysis (PCA) showing differences within (**A**) *Devaleraea mollis* and (**B**) *Palmaria hecatensis* based on their metabolite profiles in response to either VSE, F/2, or JS (See Table S2 for statistical outputs).

In addition to the differences between species, the PCA also revealed separation within species in response to the nutrient source treatments (Figure 2). VSE, F/2, and JS formed distinct groups for both species with no apparent overlap (Figure 2). The t-test analysis showed significant differences in the expression of metabolites linked to energy metabolism, amino acid and protein synthesis, nucleotide and DNA/RNA metabolism, production of secondary metabolites, and cell growth metabolites (Figures S3 and S4, Table 2). In addition, *P. hecatensis* also expressed significant differences in carbohydrate metabolism as a function of nutrient source (Figure S4, Table 3).

In general, the expression of metabolites driving significant differences between treatments was higher in *P. hecatensis* (i.e., 25; Table 3) than *D. mollis* (i.e., 15; Table 2), while the magnitude of expression differed depending on the nutrient source (Figures S3 and S4). Specifically, VSE seemed to favor energy metabolism for both species, although the metabolites showing significant differences were species-specific (Figures S3 and S4; Table 2 and Table 3). Amino acid metabolism and protein synthesis were significantly favored with the addition of JS compared to VSE and F/2 when used to grow *D. mollis* and *P. hecatensis* (Figures S3 and S4; Table 2 and Table 3). However, VSE and F/2 also prompted the increased expression of 1-Aminocyclopropanecarboxylate, N-Acetylserine, proline, and N-Acetylleucine, also linked to protein regulation and stress responses in *P. hecatensis* (Figure S4; Table 3).

Jack’s Special (JS) was the only nutrient source driving significant differences in the expression of metabolites linked to nucleotide and DNA/RNA, as well as cell growth for both species (Table 2 and Table 3). The expression of secondary metabolites differed by species and nutrient source, with a somewhat unresolved pattern (Table 2 and Table 3). Lastly, only *P. hecatensis* grown with JS showed significantly higher expression of 2-acetamido-2-deoxy-beta-D-glucosamine, a metabolite linked to carbohydrate metabolism (Figure S4, Table 3).

## 4. Discussion

This study revealed distinct metabolic profiles and responses to different nitrogen formulations in *D. mollis* and *P. hecatensis*. On the one hand, *D. mollis* favored rapid growth and energy storage, while on the other, *P. hecatensis* favored nitrogen use optimization for protein synthesis. Results support the hypothesis that these closely related species exhibit divergent metabolic performance and highlight the unique metabolic strategies each has evolved. Differences are likely linked to adaptations to the ecological or environmental conditions the species are exposed to, consistent with previous research showing differences in growth rates and physiological responses between the species [8,9].

Overall, *D. mollis* shows a greater capacity for energy storage and a higher concentration of metabolites related to energy production, suggesting higher metabolic activity and energy demands, possibly due to its faster growth rates [29]. Additionally, *D. mollis* demonstrated increased DNA/RNA synthesis and greater expression of metabolites involved in cell division, indicating more active cellular machinery, possibly related to relatively high growth rates. Jung et al. [10] further support this by reporting that *D. mollis* exhibits higher specific growth rates than *P. hecatensis*, regardless of nitrogen source. In their study, authors found at least a 2% difference in average daily growth rates, with *D. mollis* growing up to approximately 12% per day. Aside from metabolites linked to cell division, elevated levels of secondary metabolites related to DNA synthesis, repair, and light sensing suggest that *D. mollis* has evolved enhanced mechanisms to maintain genomic integrity and thrive in variable environmental conditions, possibly explaining its broader ecological adaptability and wider geographical range compared to *P. hecatensis* [24].

In contrast, *P. hecatensis* exhibited higher expression of metabolites associated with amino acid metabolism and protein synthesis, indicating a metabolic strategy focused on efficient nitrogen assimilation and protein synthesis [30,31]. This suggests *P. hecatensis* may prioritize protein production [32], potentially making it better suited to environments where rapid protein turnover or synthesis is critical, such as areas with lower light availability, colder temperatures, or limited nutrients [15]. These results align with personal observations in areas where both species coexist, as *P. hecatensis* standing biomass typically surpasses that of *D. mollis* along the Alaskan coast. At the same time, *D. mollis* is more common further south along the coast. The focus on protein synthesis may also explain *P. hecatensis’s* dominance in areas influenced by glacial discharge, where low temperatures, high turbidity, and variable nutrients are common [9].

The higher protein synthesis capacity in *P. hecatensis* may also indicate a greater cellular maintenance and repair investment, potentially linked to its thicker, more leathery thallus than *D. mollis* [9]. This trait could account for *P. hecatensis’s* distinct growth pattern, characterized by unbranched thalli with less proliferation and slower growth compared to *D. mollis*, which seems to emphasize rapid growth and energy storage, as highlighted by our results [8]. Nitrogen sources like nitrate and ammonium significantly influence metabolite expression in seaweed due to their different assimilation pathways, energy demands, and effects on metabolism. Nitrate drives a more energy-intensive process, increasing the expression of metabolites linked to energy metabolism, stress responses, and secondary metabolite production [28,33]. Ammonium, on the other hand, promotes faster nitrogen assimilation, facilitating amino acid and protein synthesis, leading to higher growth rates and increased metabolites associated with cell growth, such as amino acids and polyamines [16].

Jack’s Special (JS) primarily drove the significant differences in metabolite expression between *D. mollis* and *P. hecatensis*. In *D. mollis*, JS (nitrate and ammonium) promoted higher creatine production, indicating enhanced energy storage and regeneration to meet rapid energy demands and support cellular maintenance [34,35]. Under VSE (nitrate-only), *D. mollis* showed elevated levels of alpha-hydroxyisobutyrate and glycerol, suggesting nitrate alone stimulates energy production and osmoregulation pathways [36,37]. In *P. hecatensis*, VSE increased alpha-hydroxyisobutyrate, glycerol, and succinate, suggesting that nitrate stimulates energy production through amino acid breakdown and enhanced citric acid cycle activity [38]. This implies *P. hecatensis* relies more on these pathways when nitrate is the sole nitrogen source, while *D. mollis* shows greater flexibility, balancing energy storage and stress regulation depending on nutrient availability [24].

Furthermore, in *D. mollis*, JS significantly enhanced metabolites related to polyamine metabolism, cell growth, nucleotide and DNA/RNA metabolism, and amino acid metabolism, suggesting a focus on rapid cell division and protein synthesis under combined nitrate and ammonium conditions, leading to enhanced growth and metabolic activity [39]. In *P. hecatensis*, JS also enhanced polyamine metabolism, cell growth, and nucleotide metabolism but favored carbohydrate metabolism, suggesting a focus on energy production and storage through carbohydrate pathways while still supporting growth [32]. The amino acid metabolism response to JS in *P. hecatensis* was mixed. While JS promoted metabolites such as citrulline, glutamine, histidinol, isoleucine, N-acetylproline, ornithine, and leucine, VSE favored 1-aminocyclopropanecarboxylate and N-acetylserine, and F/2 (nitrate-only) stimulated proline and N-acetylleucine. This variation highlights how each nitrogen source uniquely modulates amino acid metabolism, with JS supporting broader amino acid production, while VSE and F/2 drive the synthesis of specific metabolites.

These findings emphasize species-specific metabolic adaptations to nitrogen formulations, illustrating how *D. mollis* and *P. hecatensis* prioritize different metabolic processes based on nitrogen availability [2,15]. A similar trend was observed in the expression of metabolites linked to secondary metabolism and signaling molecules in both species. The choice of nitrogen source plays a critical role in shaping seaweed metabolic profiles, influencing growth, stress responses, and bioactive compound production. Understanding these effects can help optimize nutrient formulations in seaweed cultivation, enhancing desired traits like improved growth rates, higher protein content, or increased production of bioactive compounds.

However, this study also faces limitations. Each species has a unique metabolome shaped by its evolutionary history and environment, making it difficult to generalize findings across species and populations. Some metabolites may be present in one species and absent in another, limiting the scope of cross-species comparisons. Additionally, incomplete metabolite databases, particularly for non-model organisms like *D. mollis* and *P. hecatensis*, pose challenges in identifying and annotating metabolites accurately. Finally, metabolomics provides only a snapshot of the metabolome, which fluctuates in response to gene expression, protein activity, and environmental factors.

To address these limitations, future studies that use metabolomics to optimize cultivation methods should integrate multi-omics approaches, combining metabolomics with transcriptomics, to capture a more comprehensive view of metabolic regulation. Expanding reference metabolomes for non-model species and improving annotation accuracy will also enhance our ability to interpret metabolomic data. This will ultimately lead to a more complete understanding of the physiological processes driving species-specific responses to environmental conditions. To the best of our knowledge, this is the first study to investigate the metabolomics of these seaweed species, and there is considerable potential expansion in future research. We view this study as an initial effort that could support future multi-omic analyses.

## 5. Conclusions

*D. mollis* and *P. hecatensis* exhibit distinct metabolic responses to nitrogen supplementation, supporting the hypothesis of divergent metabolic performance between these closely related species. *D. mollis* favors rapid growth and energy storage, while *P. hecatensis* prioritizes nitrogen optimization for protein synthesis, reflecting unique adaptations to their respective environments.

*D. mollis* showed higher concentrations of metabolites associated with energy production, DNA/RNA synthesis, and cell division, indicating higher metabolic activity and a capacity for faster growth. In contrast, *P. hecatensis* exhibited higher expression of metabolites related to amino acid metabolism and protein synthesis, suggesting a focus on efficient nitrogen assimilation and protein production.

The choice of nitrogen source, particularly Jack’s Special (JS), significantly influenced metabolite expression. *D. mollis* exhibited higher creatine levels under JS, promoting energy storage and regeneration, while *P. hecatensis* displayed increased energy production through carbohydrate pathways. Each nitrogen source uniquely modulated amino acid metabolism, with JS supporting broader amino acid production and VSE and F/2 promoting specific metabolites.

*D. mollis* appears to have evolved mechanisms to maintain genomic integrity and thrive in variable environments, explaining its broader geographical distribution and, perhaps, ecological adaptability. *P. hecatensis* seems better suited to environments with low light, cold temperatures, and limited nutrients, such as those influenced by glacial discharge, focusing on protein synthesis and cellular maintenance.

The study is limited by species-specific metabolomes, making it difficult to generalize findings across species. Incomplete metabolite databases, particularly for non-model organisms, also pose metabolite identification and annotation challenges. Metabolomics alone provides only a snapshot of the metabolome, which may not fully capture the species’ physiological responses.

## Figures and Tables

**Table 1:** Functional grouping of metabolites driving significant differences (*t*-test < 0.05) between *Devaleraea mollis* and *Palmaria hecatensis.* Arrows indicate the relative concentration trend between species.

Functional Grouping	Metabolite	*Devaleraea mollis*	*Palmaria hecatensis*
Energy metabolism	Creatine	↑	↓
	Guanidinoacetate	↑	↓
	Pantothenate	↑	↓
Amino acid metabolism	Arginine	↓	↑
and protein synthesis	Glutamine	↓	↑
	N-Acetylleucine	↓	↑
	Tryptophan	↓	↑
Nucleotide and	Dihydrouracil	↑	↓
DNA/RNA metabolism	Thymidine	↑	↓
	Uracil	↑	↓
	Uridine	↑	↓
Cell growth	4-Guanidinobutanoate	↑	↓
	N-Acetylputrescine	↑	↓
Secondary metabolites	2,6-Dihydroxypyridine	↑	↓
	3-(2-Hydroxyphenyl)propanoate	↑	↓
	4-Amino-4-Hydroxybenzoate	↑	↓
	Lumichrome	↑	↓
	Shikimate	↑	↓
	Quinate	↑	↓
Signaling molecules	3-Methoxytyramine	↓	↑
	4-Hydroxy-3-Methoxyphenylglycol	↑	↓

**Table 2:** Functional grouping of metabolites showing significant differences (*t*-test < 0.05) between VSE, F/2, and JS in *Devaleraea mollis.* Relative metabolite concentrations are ranked highest (1) to lowest (3). Metabolites that significantly differ among nutrient sources are marked in superscript with the corresponding treatment. Some metabolites are mentioned twice because they are relevant to multiple functional groupings.

Functional Grouping	*D. mollis* Metabolite	VSE	F/2	JS
Energy metabolism	Creatine	2^F/2,JS^	3^VSE,JS^	1^VSE,F/2^
	Alpha-Hydroxyisobutyrate	1^F/2,JS^	2^VSE^	3^VSE^
	Glycerol	1^F/2,JS^	2^VSE^	3^VSE^
Amino acid metabolism	Citrulline	3^JS^	2	1^VSE^
and protein synthesis	Glutamine	3^JS^	2	1^VSE^
	N-Acetylleucine	3^F/S,JS^	2^VSE^	1^VSE^
	Ornithine	3^F/2,JS^	2^VSE,JS^	1^VSE,F/2^
	Serine	3^F/2,JS^	2^VSE^	1^VSE^
Nucleotide and	Cytidine	3^F/2,JS^	2^VSE,JS^	1^VSE,F/2^
DNA/RNA metabolism	Guanine	3^F/2,JS^	2^VSE^	1^VSE^
	Hypoxanthine	3^F/2,JS^	2^VSE^	1^VSE^
	Thymidine	3^F/2,JS^	2^VSE,JS^	1^VSE,F/2^
Cell growth	4-Guanidinobutanoate	3^F/2,JS^	2^VSE,JS^	1^VSE,F/2^
	Ornithine	3^F/2,JS^	2^VSE,JS^	1^VSE,F/2^
Secondary metabolites	2,6-Dihydroxypyridine	1^F/2,JS^	3^VSE^	2^VSE^
	Trigonelline	2^F/2^	1^VSE,JS^	3^F/2^

**Table 3:** Functional grouping of metabolites showing significant differences (*t*-test < 0.05) between VSE, F/2, and JS in *Palmaria hecatensis.* Relative metabolite concentrations are ranked highest (1) to lowest (3). Metabolites that significantly differ among nutrient sources are marked in superscript with the corresponding treatment. Some metabolites are mentioned twice because they are relevant to multiple functional groupings.

Functional Grouping	*P. hecatensis* Metabolite	VSE	F/2	JS
Energy metabolism	Alpha-Hydroxyisobutyrate	1^F/2,JS^	2^VSE,JS^	3^VSE,F/2^
	Glycerol	1^F/2,JS^	2^VSE^	3^VSE^
	Succinate	1^F/2,JS^	2^VSE^	3^VSE^
Amino acid metabolism	1-Aminocyclopropanecarboxylate	1^F/2,JS^	2^VSE^	3^VSE^
and protein synthesis	Citrulline	2^JS^	3^JS^	1^VSE,F/2^
	Glutamine	3^F/2,JS^	2^VSE,JS^	1^VSE,F/2^
	Histidinol	3^F/2,JS^	2^VSE^	1^VSE^
	Isoleucine	2^JS^	3^JS^	1^VSE,F/2^
	Leucine	2^F/2,JS^	3^VSE,JS^	1^VSE,F/2^
	N-Acetylleucine	3^F/2,JS^	1^VSE^	2^VSE^
	N-Acetylproline	3^JS^	2^JS^	1^VSE,F/2^
	N-Acetylserine	1^F/2,JS^	2^VSE,JS^	3^VSE,F/2^
	Ornithine	2^JS^	3^JS^	1^VSE,F/2^
	Proline	2^F/2^	1^VSE,JS^	3^F/2^
Nucleotide and	Cytidine	3^JS^	2	1^VSE^
DNA/RNA metabolism	Dihydrouracil	2^F/2^	3^VSE,JS^	1^F/2^
	Uridine	2^JS^	3^JS^	1^VSE,F/2^
Cell growth	N-Acetylputrescine	3^F/2,JS^	2^VSE,JS^	1^VSE,F/2^
	Ornithine	2^JS^	3^JS^	1^VSE,F/2^
Secondary metabolites	3-Methoxytyramine	1^F/2,JS^	2^VSE,JS^	3^VSE,F/2^
	Porphobilinogen	3^F/2,JS^	2^VSE,JS^	1^VSE,F/2^
	Riboflavin	3^JS^	2^JS^	1^VSE,F/2^
Signaling molecules	3-Methoxytyramine	1^F/2,JS^	2^VSE,JS^	3^VSE,F/2^
	Nicotinamide	1^JS^	2^JS^	3^VSE,F/2^
Carbohydrate metabolism	2-Acetamido-2-Deoxy-Beta-D- Glucosamine	3^JS^	2^JS^	1^VSE,F/2^

## Data Availability

Data supporting reported results can be available upon request.

## Supplementary Materials

The following supporting information can be downloaded at: https://www.mdpi.com/article/10.3390/life15020143/s1, Figure S1: Compound discoverer; Figure S2: Metabolites, both species; Figure S3: Metabolites, *D. mollis*; Figure S4: Metabolites, *P. hecatensis*. Table S1: Enrichment Formulation, Table S2: Statistical outputs.

## References

[1] Buschmann A.H., Camus C., Infante J., Neori A., Israel Á., Hernández-González M.C., Critchley A.T. (2017). Seaweed production: Overview of the global state of exploitation, farming and emerging research activity. Eur. J. Phycol..

[2] Kotowicz D.M., Concepcion A., Bradt G., Chadsey M., Clemetson A., Good M., Reitsma J., Robidoux J. (2024). Identifying challenges of the US domestic seaweed aquaculture industry. Aquaculture.

[3] Bindu M.S., Levine I.A. (2010). The commercial red seaweed Kappaphycus alvarezii—An overview on farming and environment. J. Appl. Phycol..

[4] Loureiro R., Gachon C.M., Rebours C. (2015). Seaweed cultivation: Potential and challenges of crop domestication at an unprecedented pace. New Phytol..

[5] Tullberg R.M., Nguyen H.P., Wang C.M. (2022). Review of the status and developments in seaweed farming infrastructure. J. Mar. Sci. Eng..

[6] Demetropoulos C.L., Langdon C.J. (2004). Enhanced production of Pacific dulse (*Palmaria mollis*) for co-culture with abalone in a land-based system: Effects of stocking density, light, salinity, and temperature. Aquaculture.

[7] Saunders G.W., Jackson C., Salomaki E.D. (2018). Phylogenetic analyses of transcriptome data resolve familial assignments for genera of the red-algal Acrochaetiales-Palmariales Complex (Nemaliophycidae). Mol. Phylogenet. Evol..

[8] Dittrich M.C., Meyer L., Kelley A., Stekoll M.S.S., Umanzor S. (2024). Cultivation protocols for the rhodophytes, *Devaleraea mollis* and *Palmaria hecatensis* from Alaska. BioRxiv.

[9] Lindeberg M., Lindstrom S.C. (2010). A Field Guide to Seaweeds of Alaska.

[10] Jung J.W., Dittrich M.C., Kim J.K., Umanzor S. (2024). Exploring nutrient supplements for enhanced growth and quality of *Devaleraea mollis* and *Palmaria hecatensis*. J. Appl. Phycol..

[11] Hawkes M.W. (1985). *Palmaria hecatensis* sp. nov. (Rhodophyta, *Palmariales*) from British Columbia and Alaska with a survey of other *Palmaria* species. Can. J. Bot..

[12] Shah N.J., Sureshkumar S., Shewade D.G. (2015). Metabolomics: A tool ahead for understanding molecular mechanisms of drugs and diseases. Indian J. Clin. Biochem..

[13] Roleda M.Y., Hurd C.L. (2019). Seaweed nutrient physiology: Application of concepts to aquaculture and bioremediation. Phycologia.

[14] Robertson-Andersson D.V., McKenzie L.J. (2008). The effect of different nitrogen sources on the growth and biochemical composition of *Gracilaria tenuistipitata*. Aquat. Bot..

[15] Wang Q., Lan L., Li H., Gong Q., Gao X. (2023). Effects of nitrogen source and concentration on the growth and biochemical composition of the red seaweed *Grateloupia turuturu* (Halymeniaceae, Rhodophyta). Sustainability.

[16] Dawes C.J., Koch E.W. (1990). Physiological responses of the red algae Gracilaria verrucosa and G. tikvahiae before and after nutrient enrichment. Bull. Mar. Sci..

[17] Morgan K.C., Wright J.L.C., Simpson F.J. (1980). Review of chemical constituents of the red alga *Palmaria palmata* (dulse). Econ. Bot..

[18] Mouritsen O.G., Dawczynski C., Duelund L., Jensen H.M., Sommer H.M., Olsen K. (2013). On the human consumption of the red seaweed dulse (*Palmaria palmata* (L.) Weber & Mohr). J. Appl. Phycol..

[19] Lalegerie F., Stiger-Pouvreau V., Connan S. (2024). Mycosporine-like Amino Acids in *Palmaria palmata* (Rhodophyta): Specific Implication of Usujirene in Photoprotection. Mar. Drugs.

[20] Kim J.K., Yarish C. (2004). Development of a sustainable land-based seaweed aquaculture system suitable for the Northeast US. Bull. Korean Fish. Soc..

[21] Friedlander M., Dawes C.J. (1985). In situ uptake kinetics of ammonium and phosphate and chemical composition of the red seaweed *Gracilaria tikvahiae*. J. Phycol..

[22] Idowu A.T., Amigo-Benavent M., Santos-Hernández M., Tiwari B., Hayes M. (2023). Impact of growth conditions on the nitrogen, protein, colour, and amino acid profiles of the cultured macroalga, *Palmaria palmata*. J. Appl. Phycol..

[23] Rosen G., Langdon C.J., Evans F. (2000). The nutritional value of *Palmaria mollis* cultured under different light intensities and water exchange rates for juvenile red abalone *Haliotis rufescens*. Aquaculture.

[24] Skriptsova A.V., Shibneva S., Semenchenko A.A. (2023). Morphological and molecular investigations shed light on diversity and distribution of Palmariaceae in the north-western Pacific. Eur. J. Phycol..

[25] Redmond S., Green L., Yarish C., Kim J.K., Neefus C. (2014). New England Seaweed Culture Handbook: Nursery Systems.

[26] Werner A., Dring M.J., Wiencke B., Bischof K. (2011). Cultivation of *Palmaria palmata* (dulse). Seaweed Biology.

[27] Guillard R.R.L., Smith W.L., Chanley M.H. (1975). Culture of phytoplankton for feeding marine invertebrates. Culture of Marine Invertebrate Animals.

[28] Schymanski E.L., Jeon J., Gulde R., Fenner K., Ruff M., Singer H.P., Hollender J. (2014). Identifying small molecules via high resolution mass spectrometry: Communicating confidence. Environ. Sci. Technol..

[29] Hurd C., Harrison P., Bischof K., Lobban C. (2014). Seaweed Ecology and Physiology.

[30] Lobban C.S., Harrison P.J. (1994). Seaweed Ecology and Physiology.

[31] Xu G., Fan X., Miller A.J. (2012). Plant Nitrogen Assimilation and Use Efficiency. Front. Plant Sci..

[32] Stévant P., Rebours C., Chapman A. (2017). Seaweed aquaculture in Norway: Recent industrial developments and future perspectives. Aquac. Int..

[33] Harrison P.J., Druehl L.D., Lloyd K.E., Thompson P.A. (1986). Nitrogen uptake kinetics in three species of laminarian kelp as a function of dissolved nitrogen concentration. Mar. Biol..

[34] Bidwell R.G.S., McLachlan J. (1985). Carbon nutrition of seaweeds: Photosynthesis and growth. J. Exp. Mar. Biol. Ecol..

[35] Thomas T.E., Turpin D.H. (1980). Desiccation-enhanced nutrient uptake rates in the intertidal alga *Fucus distichus*. Bot. Mar..

[36] Davison I.R., Pearson G.A. (1996). Stress tolerance in intertidal seaweeds. J. Phycol..

[37] Falkowski P.G., Raven J.A. (2007). Aquatic Photosynthesis.

[38] Levitt J. (1980). Responses of Plants to Environmental Stresses: Water, Radiation, Salt, and Other Stresses.

[39] Buchanan B.B., Gruissem W., Jones R.L. (2015). Biochemistry & Molecular Biology of Plants.

