# Supplementary material for: Divergent Patterns of Metabolite Expression in Red Seaweeds (Devaleraea mollis and Palmaria hecatensis) Following Nitrate and Ammonium Supplementation
Source: Life (Basel). 2025 Jan 21;15(2):143. doi: 10.3390/life15020143 (PMC11856563; doi:10.3390/life15020143)
Supplement: Supplementary file 1 [file life-15-00143-s001.zip › life-3366397-supplementary.pdf]

a)

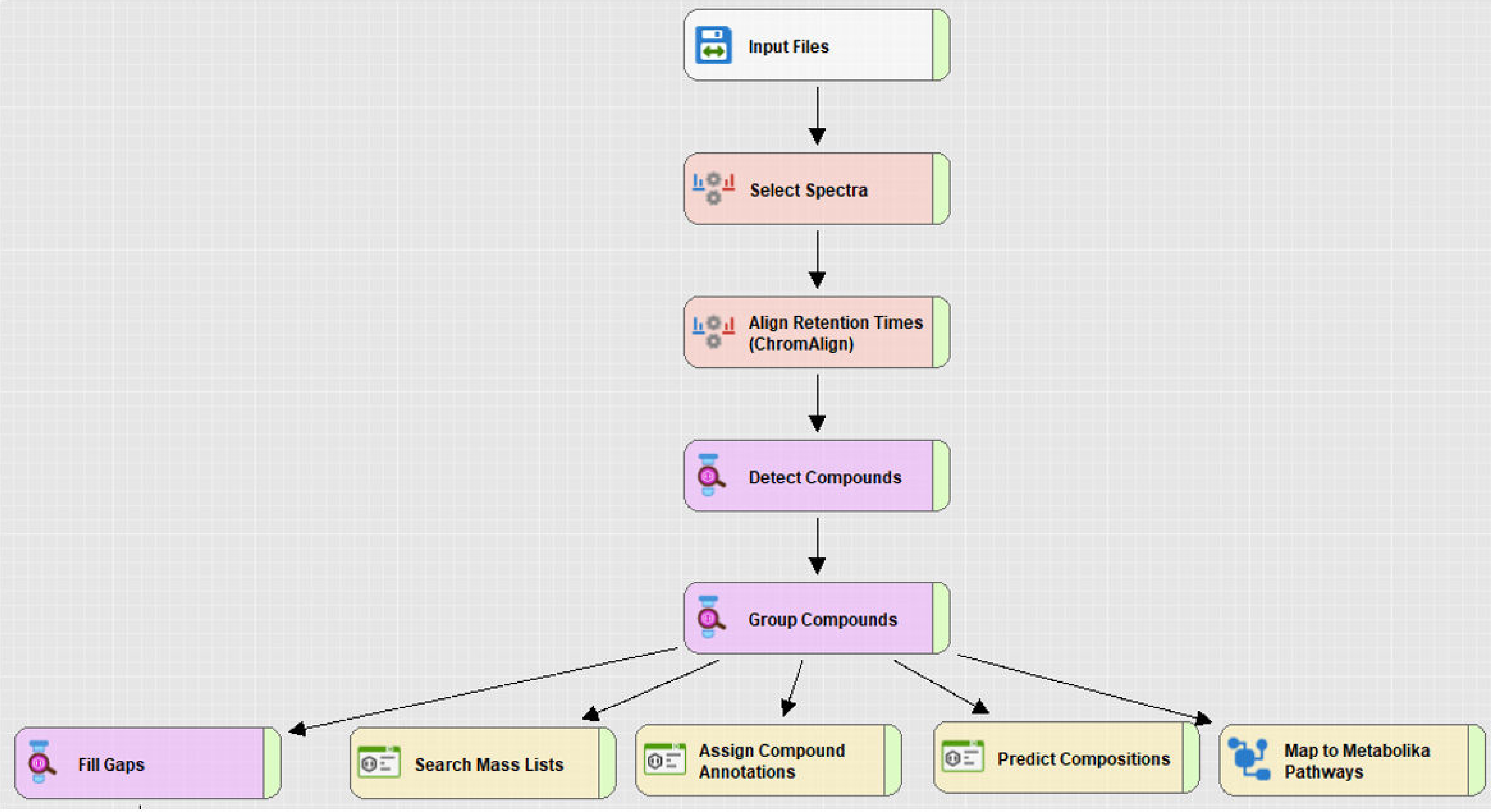

Input Files
Select Spectra
Align Retention Times (ChromAlign)
Detect Compounds
Group Compounds
Fill Gaps
Search Mass Lists
Assign Compound Annotations
Predict Compositions
Map to Metabolika Pathways

b)

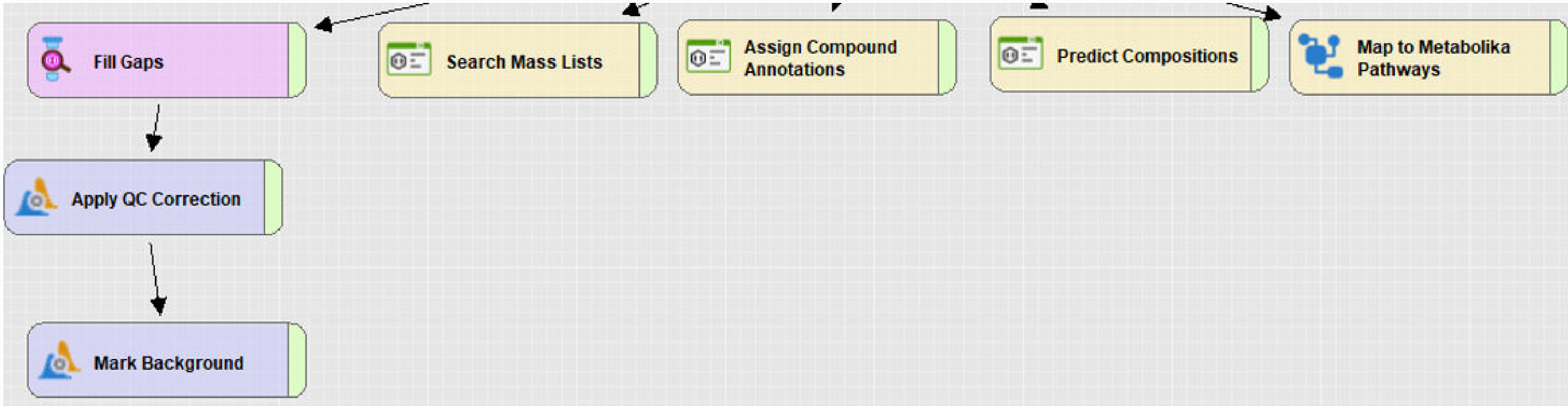

Fill Gaps
Search Mass Lists
Assign Compound Annotations
Predict Compositions
Map to Metabolika Pathways
Apply QC Correction
Mark Background

Figure S1. Original and normalized concentrations of metabolites driving significant differences (t-test < 0.05) between *Devaleraea mollis* and *Palmaria hecatensis*.

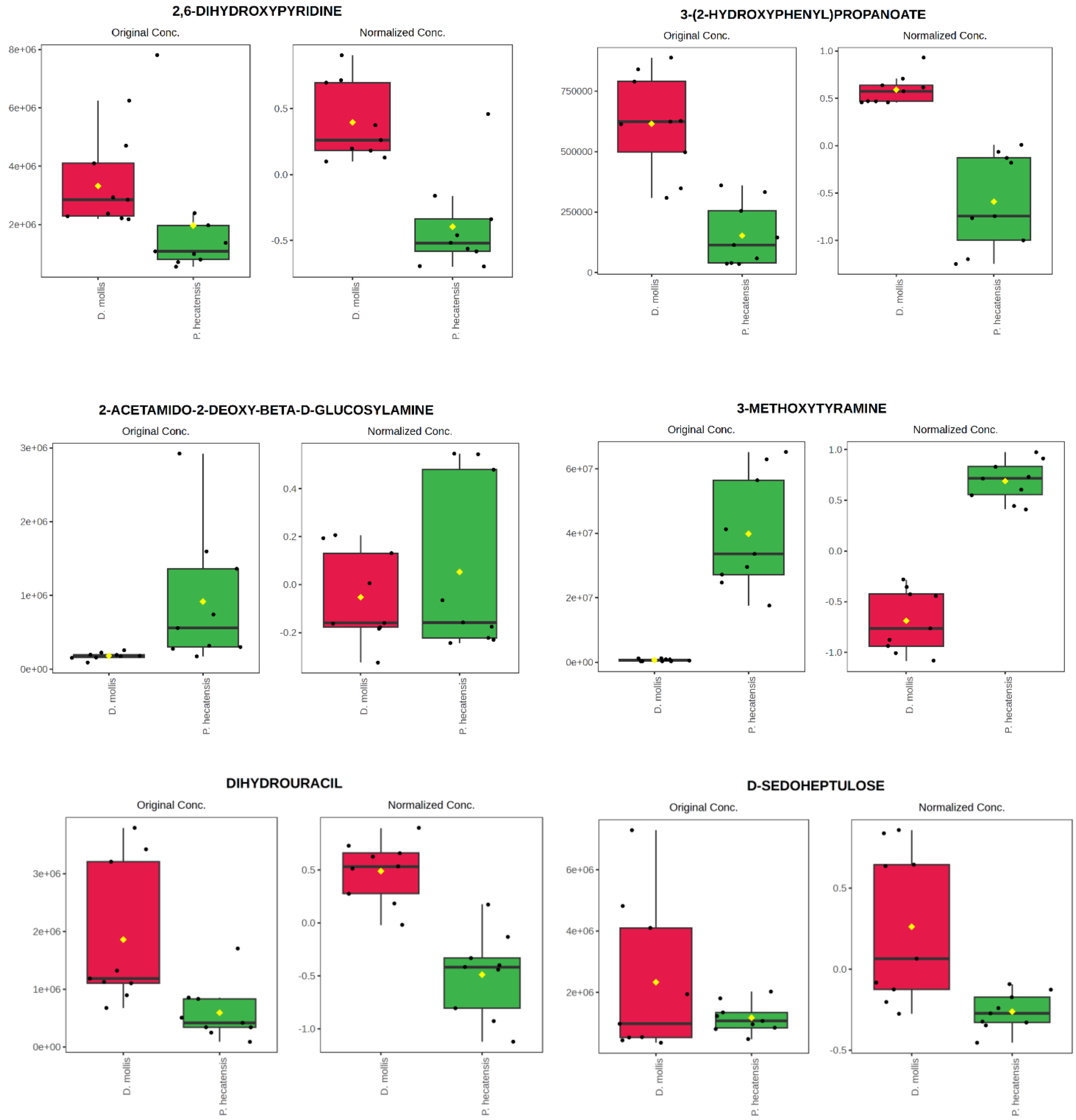

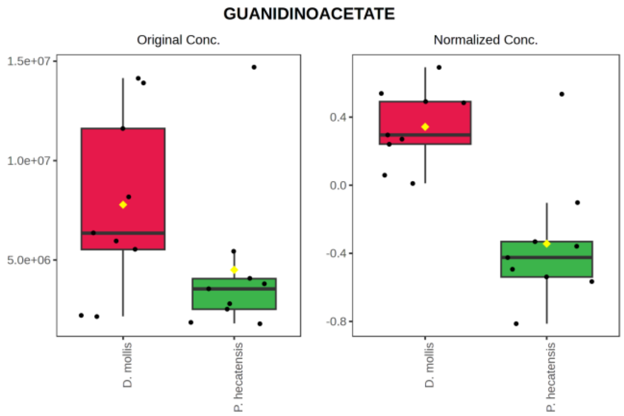
GUANIDINOACETATE
Original Conc.
Normalized Conc.
1.5e+07
1.0e+07
5.0e+06
0.4
0.0
-0.4
-0.8
D. mollis
P. hecatensis

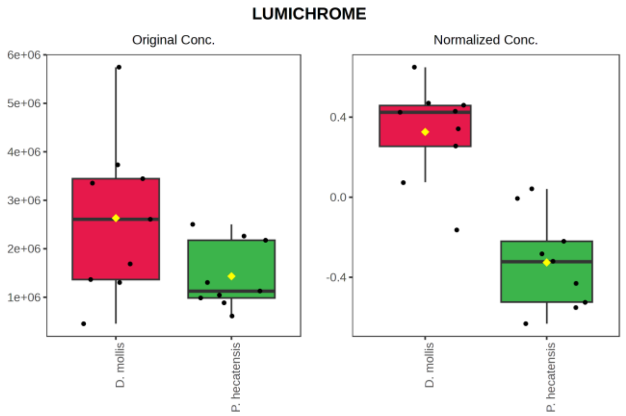
LUMICHROME
Original Conc.
Normalized Conc.
6e+06
5e+06
4e+06
3e+06
2e+06
1e+06
0.4
0.0
-0.4
D. mollis
P. hecatensis

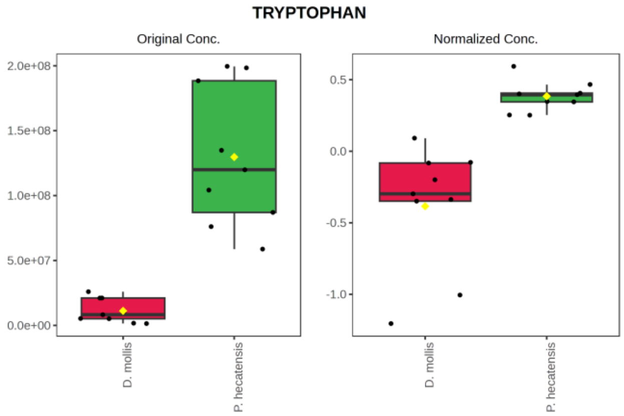
TRYPTOPHAN
Original Conc.
Normalized Conc.
2.0e+08
1.5e+08
1.0e+08
5.0e+07
0.0e+00
0.5
0.0
-0.5
-1.0
D. mollis
P. hecatensis

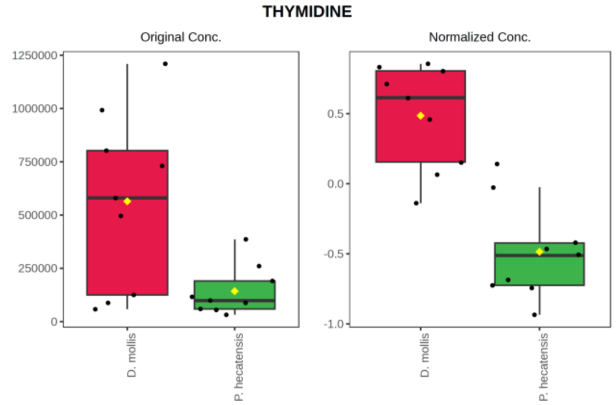
THYMIDINE
Original Conc.
Normalized Conc.
1250000
1000000
750000
500000
250000
0
0.5
0.0
-0.5
-1.0
D. mollis
P. hecatensis

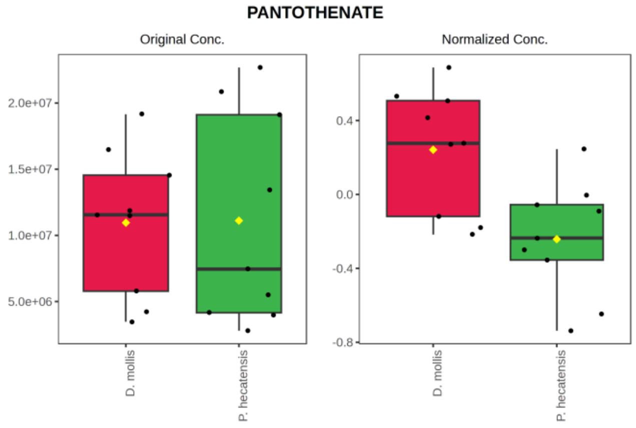
PANTOTHENATE
Original Conc.
Normalized Conc.
2.0e+07
1.5e+07
1.0e+07
5.0e+06
0.4
0.0
-0.4
-0.8
D. mollis
P. hecatensis

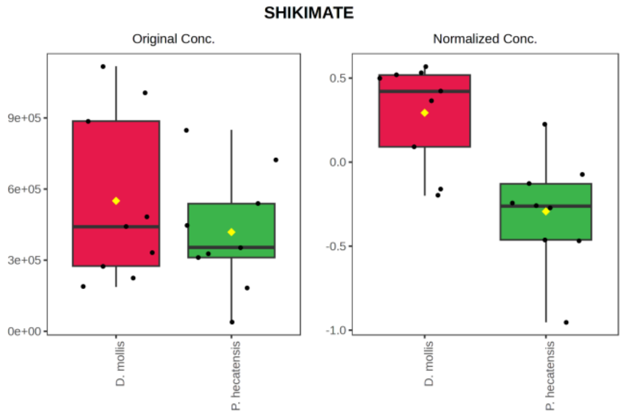
SHIKIMATE
Original Conc.
Normalized Conc.
9e+05
6e+05
3e+05
0e+00
0.5
0.0
-0.5
-1.0
D. mollis
P. hecatensis

Figure S2. Original and normalized concentrations of metabolites driving significant differences (t-test < 0.05) between nutrient source treatment in *Devaleraea mollis*.

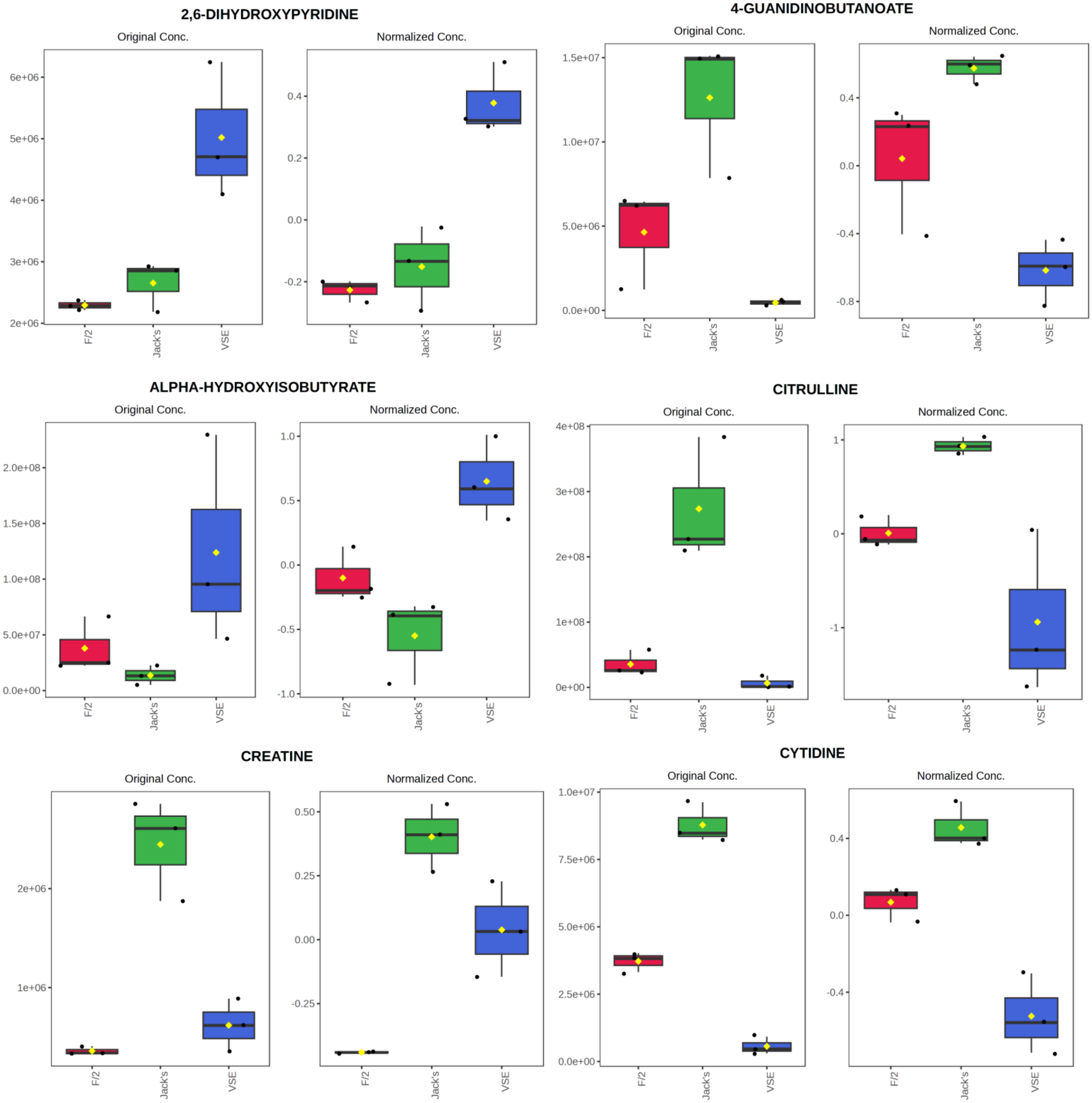

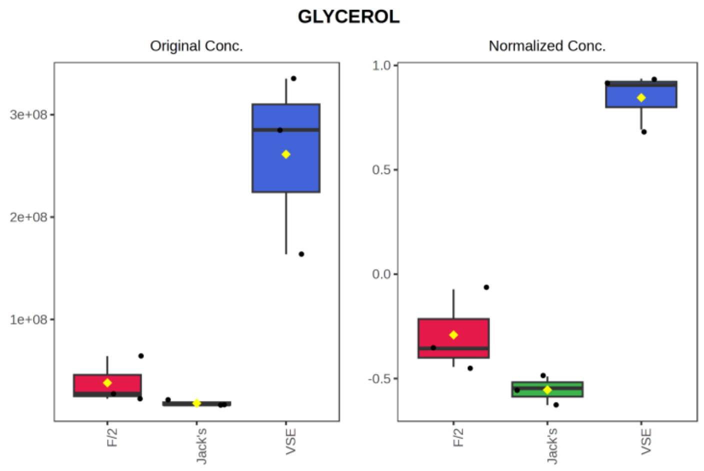
GLYCEROL
Original Conc.
Normalized Conc.
3e+08
2e+08
1e+08
1.0
0.5
0.0
-0.5
F/2
Jack's
VSE

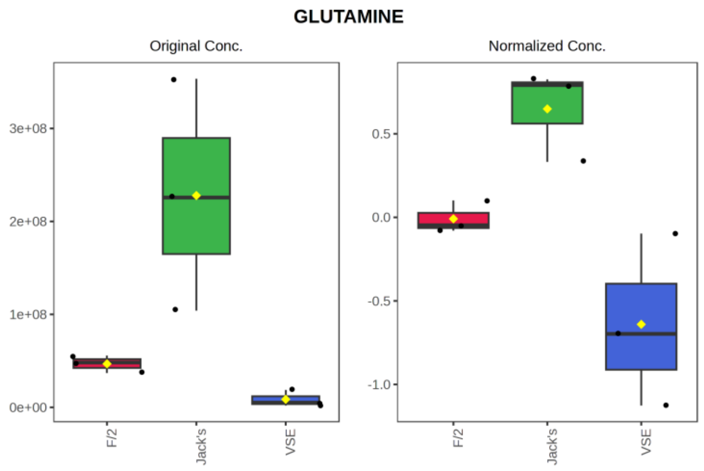
GLUTAMINE
Original Conc.
Normalized Conc.
3e+08
2e+08
1e+08
0e+00
0.5
0.0
-0.5
-1.0
F/2
Jack's
VSE

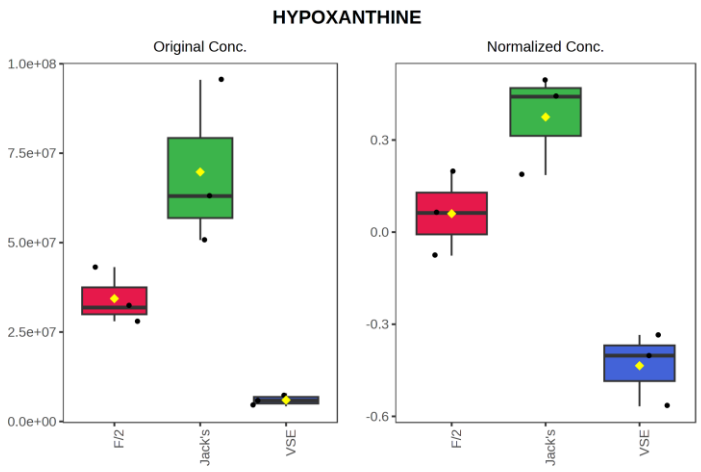
HYPOXANTHINE
Original Conc.
Normalized Conc.
1.0e+08
7.5e+07
5.0e+07
2.5e+07
0.0e+00
0.3
0.0
-0.3
-0.6
F/2
Jack's
VSE

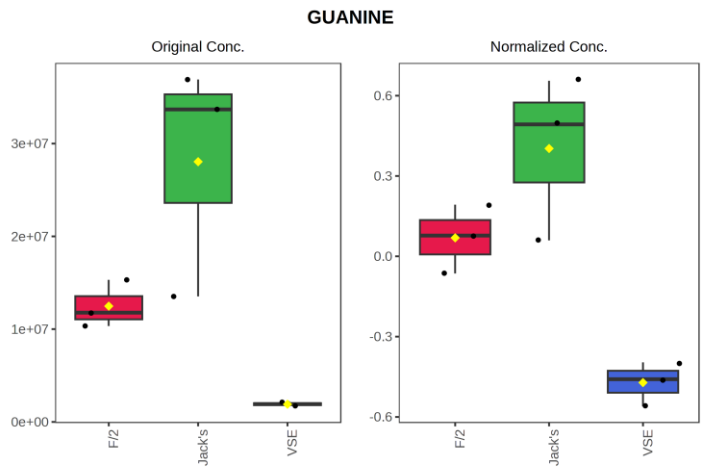
GUANINE
Original Conc.
Normalized Conc.
3e+07
2e+07
1e+07
0e+00
0.6
0.3
0.0
-0.3
-0.6
F/2
Jack's
VSE

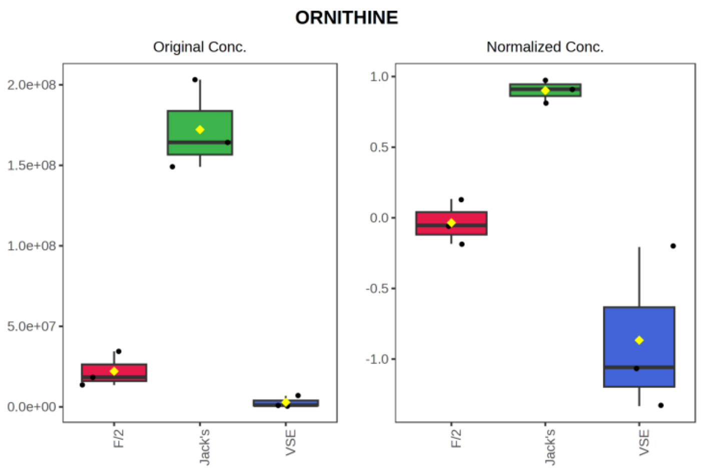
ORNITHINE
Original Conc.
Normalized Conc.
2.0e+08
1.5e+08
1.0e+08
5.0e+07
0.0e+00
1.0
0.5
0.0
-0.5
-1.0
F/2
Jack's
VSE

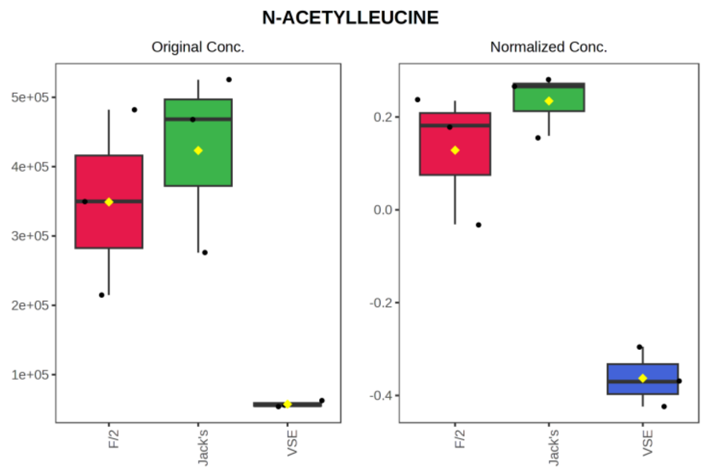
N-ACETYLLEUCINE
Original Conc.
Normalized Conc.
5e+05
4e+05
3e+05
2e+05
1e+05
0.2
0.0
-0.2
-0.4
F/2
Jack's
VSE

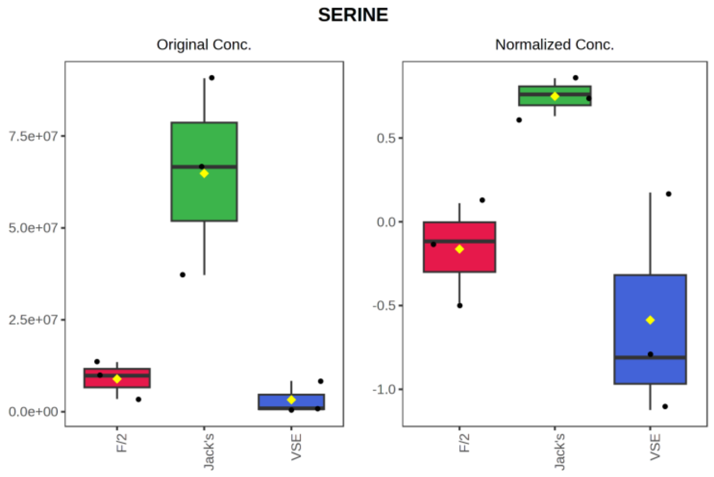
SERINE
Original Conc.
Normalized Conc.
7.5e+07
5.0e+07
2.5e+07
0.0e+00
0.5
0.0
-0.5
-1.0
F/2
Jack's
VSE

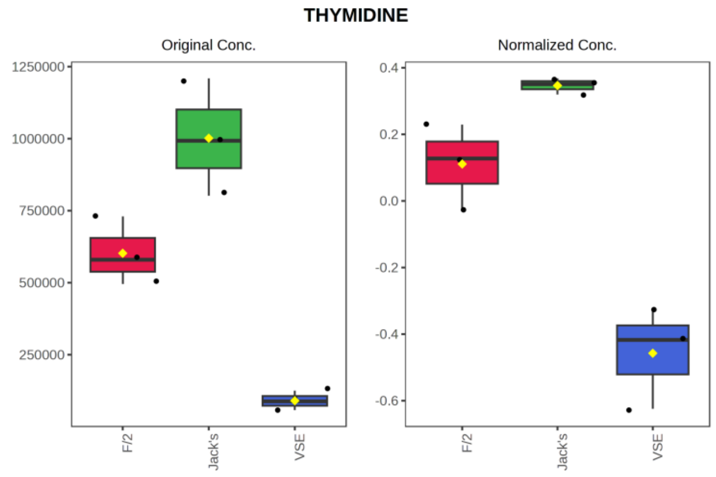
THYMIDINE
Original Conc.
Normalized Conc.
1250000
1000000
750000
500000
250000
0.4
0.2
0.0
-0.2
-0.4
-0.6
F/2
Jack's
VSE

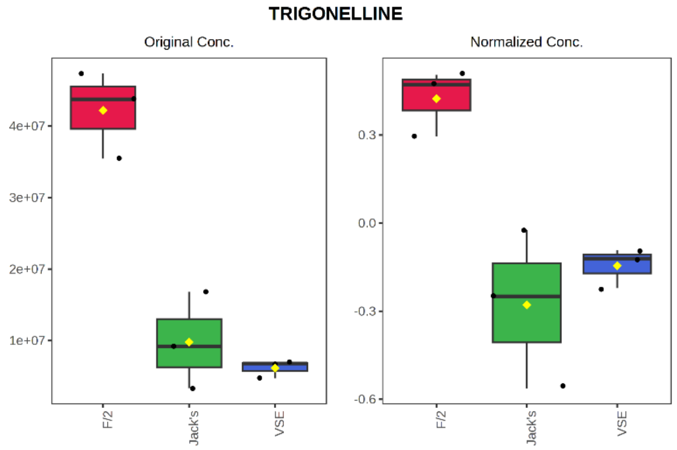
TRIGONELLINE
Original Conc.
Normalized Conc.
4e+07
3e+07
2e+07
1e+07
0.3
0.0
-0.3
-0.6
F/2
Jack's
VSE

Figure S3. Original and normalized concentrations of metabolites driving significant differences (t-test < 0.05) between nutrient source treatment in *Palmaria hecatensis*.

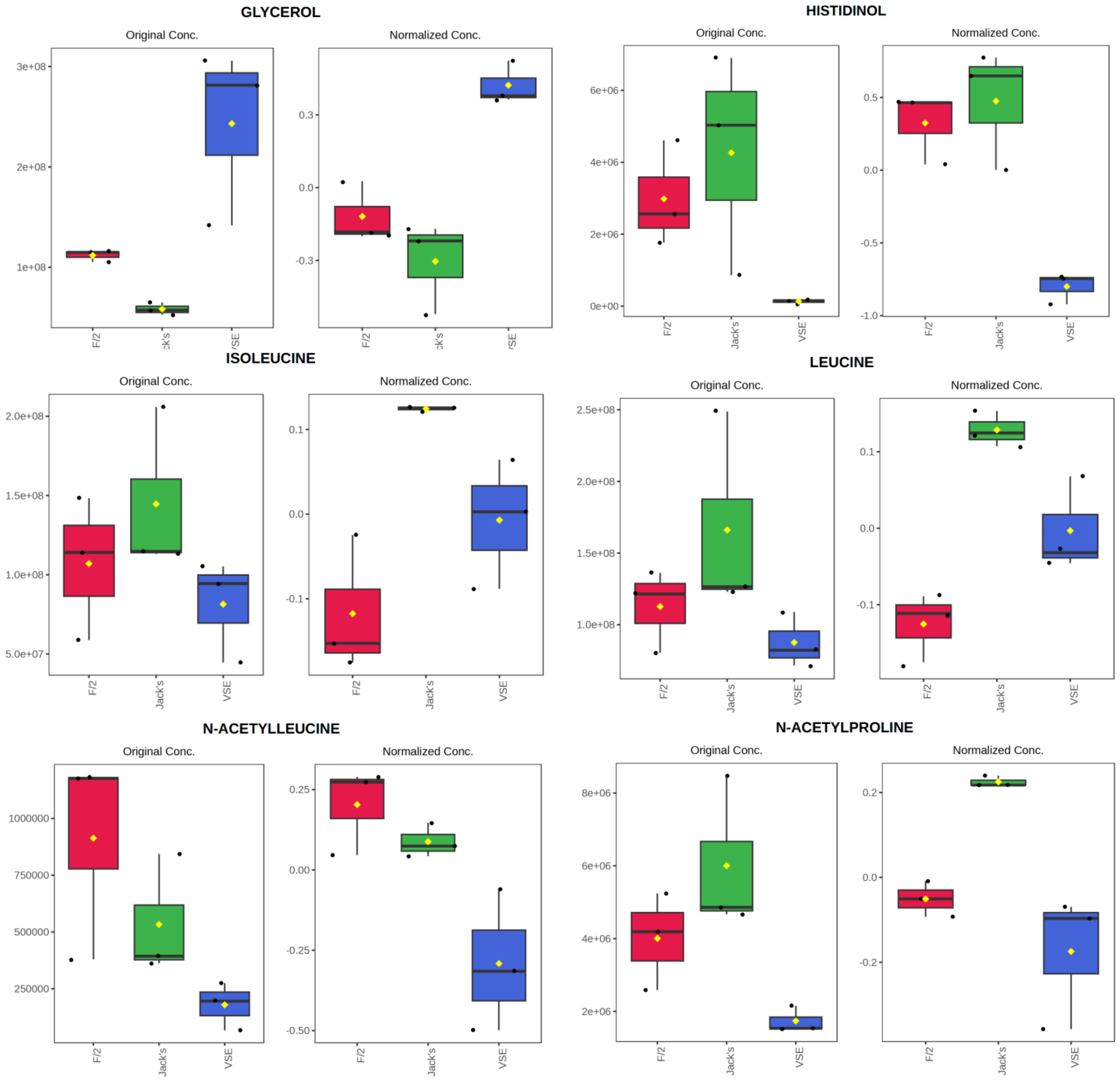

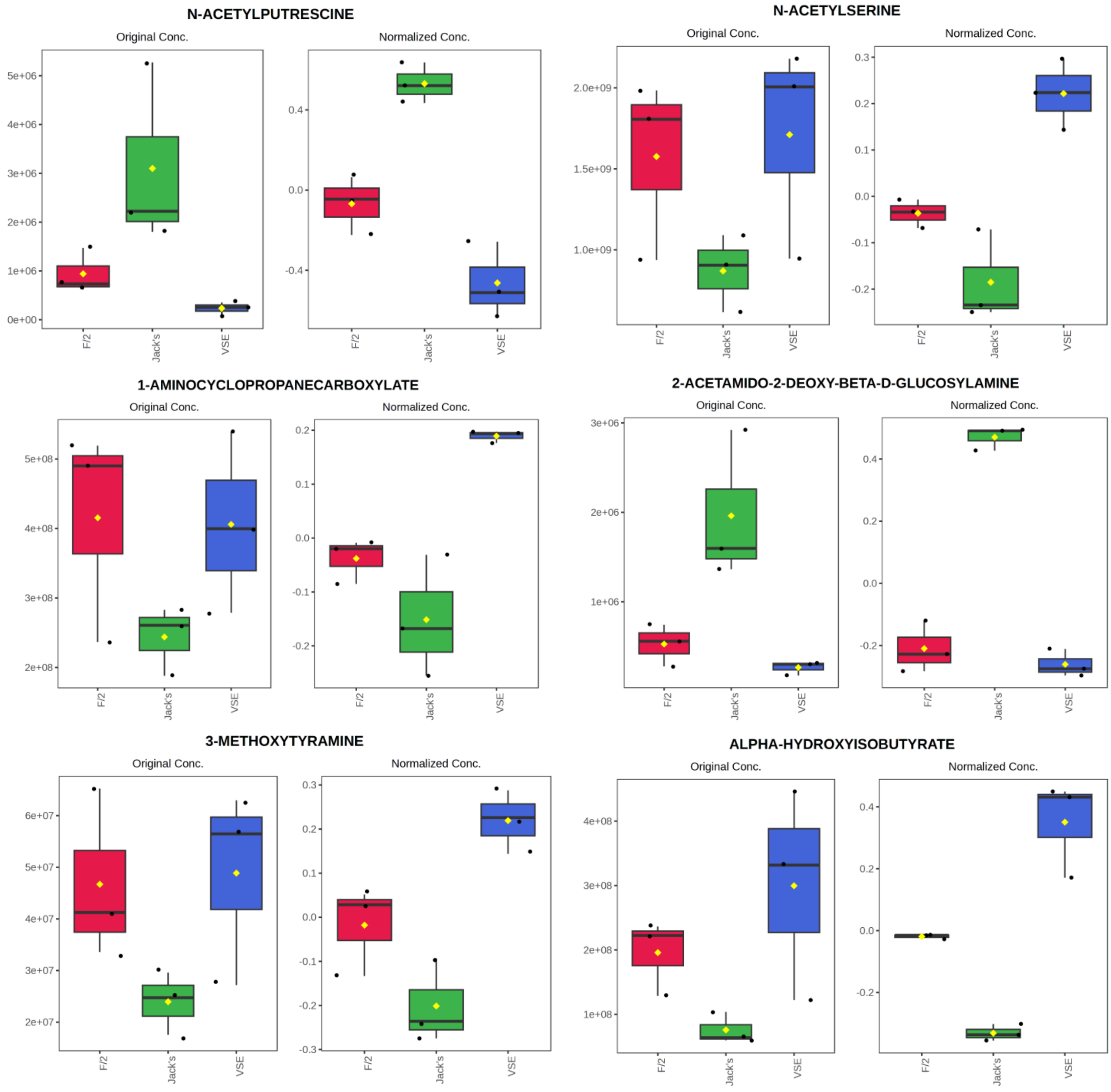

N-ACETYLPUTRESCINE
Original Conc.
Normalized Conc.
5e+06
4e+06
3e+06
2e+06
1e+06
0e+00
0.4
0.0
-0.4
F/2
Jack's
VSE
N-ACETYLSERINE
Original Conc.
Normalized Conc.
2.0e+09
1.5e+09
1.0e+09
0.3
0.2
0.1
0.0
-0.1
-0.2
1-AMINOCYCLOPROPANECARBOXYLATE
Original Conc.
Normalized Conc.
5e+08
4e+08
3e+08
2e+08
0.2
0.1
0.0
-0.1
-0.2
2-ACETAMIDO-2-DEOXY-BETA-D-GLUCOSYLAMINE
Original Conc.
Normalized Conc.
3e+06
2e+06
1e+06
0.4
0.2
0.0
-0.2
3-METHOXYTYRAMINE
Original Conc.
Normalized Conc.
6e+07
5e+07
4e+07
3e+07
2e+07
0.3
0.2
0.1
0.0
-0.1
-0.2
-0.3
ALPHA-HYDROXYISOBUTYRATE
Original Conc.
Normalized Conc.
4e+08
3e+08
2e+08
1e+08
0.4
0.2
0.0
-0.2

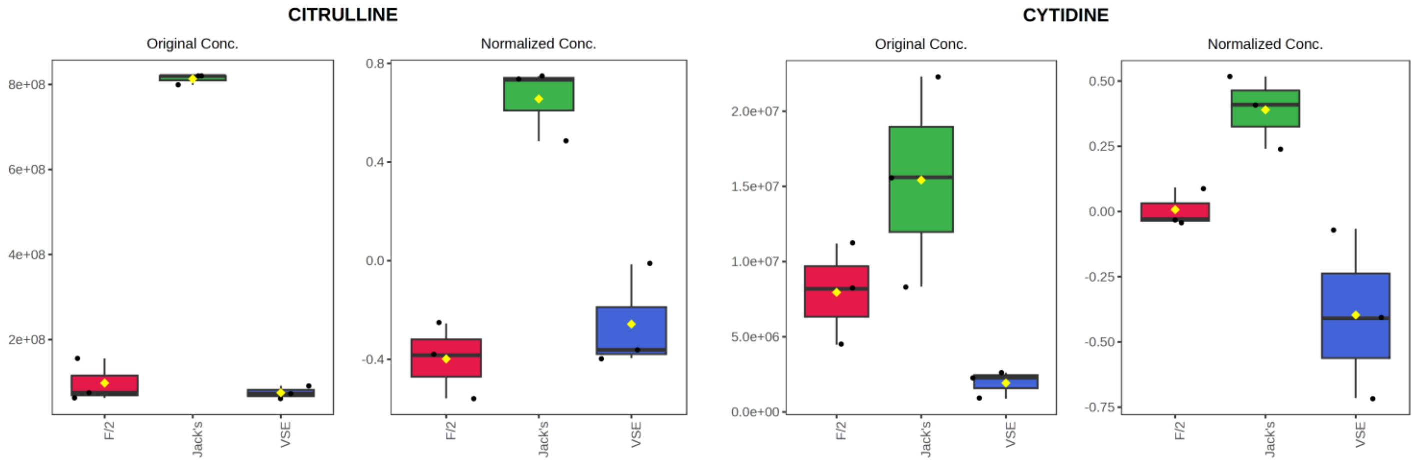
CITRULLINE
Original Conc.
Normalized Conc.
CYTIDINE
Original Conc.
Normalized Conc.
8e+08
6e+08
4e+08
2e+08
0.8
0.4
0.0
-0.4
2.0e+07
1.5e+07
1.0e+07
5.0e+06
0.0e+00
0.50
0.25
0.00
-0.25
-0.50
-0.75
F/2
Jack's
VSE

Table S1. Elemental formulation per experimental treatment. Nitrogen and Phosphorus were analyzed on every working solution while concentrations for all other constituents derived from the manufacturer's label or were calculate based on the recipe followed.

**Nutrient (µM)**	**VSE**	**F/2**	**JS**
**Nitrogen**	500 (100% NO_3^-)	500 (100% NO_3^-)	500 (57% NO_3^- and 43% NH_4^+)
**Phosphorus**	30	15	45
**Iron**	1	11.7	0.6
**EDTA**	10	11.7	-
**Boron**	-	-	0.6
**Copper**	-	0.04	0.05
**Potassium**	-	-	107
**Manganese**	10	0.9	0.3
**Magnesium**	-	-	1.15
**Molybdenum**	-	0.03	0.03
**Zinc**	-	0.05	0.004
**Vitamins (presence/absence)**	Vitamin B_1, B_{12}, Biotin	Vitamin B_1, B_{12}, Biotin	-

Table S2: Statistical outputs for PCA and PLS-DA data

Grouping	PCA statistical validation	PLS-DA statistical validation
By species	F-value: 15.388<br>R-squared: 0.049025<br>p-value: 0.001	Components: 3<br>Accuracy: 1.0<br>R-squared: 0.9875<br>Q2: 0.95586
D. mollis by nutrient	F-value: 34.242<br>R-squared: 0.91945<br>p-value: 0.004	Components: 3<br>Accuracy: 1.0<br>R-squared: 0.98957<br>Q2: 0.82679
P. hecatensis by nutrient	F-value: 28.34<br>R-squared: 0.90428<br>p-value: 0.009	Components: 2<br>Accuracy: 1.0<br>R-squared: 0.85298<br>Q2: 0.21507
